# Prevalence of Body Dysmorphic Disorder: A Systematic Review and Meta‐Analysis

**DOI:** 10.1111/jocd.70121

**Published:** 2025-04-08

**Authors:** Luis Ángel Pérez‐Buenfil,, Martha Alejandra Morales‐Sánchez

**Affiliations:** ^1^ IMSS Bienestar Ciudad de Mexico Mexico; ^2^ Facultad de Medicina Universidad Nacional Autónoma de México Mexico City Mexico

**Keywords:** body dysmorphic disorder, meta‐analysis, prevalence, systematic review

## Abstract

**Background:**

Body dysmorphic disorder (BDD) is characterized by a preoccupation with ≥ 1 perceived defects in physical appearance, which leads to social anxiety and avoidance. This excessive focus on appearance is linked to dissatisfaction with physical features, disappointment with cosmetic treatments, or frustration from not meeting perceived beauty standards.

**Objective:**

The aim of this study was to determine the prevalence of BDD in dermatology, psychiatric, plastic surgery, general population, and world regions, as well as the prevalence of BDD using different diagnostic tools.

**Methods:**

The authors conducted a systematic review of the literature in Medline via PubMed, Embase, and Latin American databases using MeSH terms “body dysmorphic disorder” and “prevalence” with filters for original and cross‐sectional studies. Statistical analysis was performed using RStudio, and bias assessment was made using JBI Critical appraisal.

**Results:**

The overall prevalence of BDD in the general population was 17%, with higher rates in females than in males. The prevalence in plastic surgery patients was 24%. The highest prevalence of BDD was found in Latin America compared to other world regions.

**Conclusions:**

BDD is a highly prevalent disorder, and increased awareness of the disease could improve its approach in plastic surgery and other medical fields.

## 1 Introduction

Body dysmorphic disorder (BDD) is a condition characterized by a preoccupation with one or more perceived defects in physical appearance that are not observable or are considered insignificant by others. It is accompanied by compulsive behaviors that are directed at addressing these appearance concerns [1] These behaviors may include excessive mirror checking, grooming, skin picking, seeking reassurance, or frequently changing clothes. The disorder often leads to significant social anxiety and avoidance. Repetitive behaviors are performed in response to self‐perceived appearance concerns, which frequently result in seeking additional cosmetic procedures to correct perceived physical defects [2, 3]. The onset of BDD typically occurs before the age of 18, and it affects more females than males [2, 4].

Body dysmorphic disorder is reported to have a significant effect on patients' mental health, increasing the risk of suicidal thoughts almost four times, which is comparable to severe psychiatric conditions such as post‐traumatic stress disorder and major depression [5, 6].

Previous systematic reviews have reported the overall prevalence of BDD to range from 0.5% to 11%. However, measuring the prevalence of BDD is challenging due to variations in diagnostic criteria and the screening tools employed across studies. For example, the highest prevalence rates (2.9%–20.0%) are found in cosmetic/dermatology settings. Veale et al. reported prevalence rates for multiple groups, including adults in the community (1.9%), psychiatric inpatients (7.4%), psychiatric outpatients (5.8%), general cosmetic surgery (13.2%), dermatology outpatients (11.3%), and other groups [7]. Minty and Minty estimated the prevalence of BDD in the global population (0.5%–3.2%), cosmetic surgery cohorts with a large range (2.9%–5.7%), general dermatology (1.3%–5.8%), psychiatric patients (2.9%–57%), and other groups [8]. McGrath et al. reported the overall prevalence in estimates for the cosmetic/dermatology (20.0%), mental health patients (7.4%), studies in Europe (11.9%), the United States (7.3%), and Brazil (25.3%). However, they did not find significance across geographical locations. They estimated prevalence for studies that used the MINI (13.2%) and SCID (11.2%) (*n* = 17), but they did not find significance in these groups [9].

The dearth of studies measuring the impact and discrepancies among countries and BDD assessment tools necessitates an analysis of the variations in prevalence by geographical region, screening tool, and sex. The objective of this systematic review was to determine the prevalence of BDD in the general population, dermatology, plastic surgery, and psychiatric patients, while accounting for differences by sex. Additionally, this study introduces the prevalence of BDD by continent and its prevalence according to various BDD screening tools for the first time.

The objective of this review was to furnish evidence that supports and encourages the integration of psychiatric assessments into general dermatology practice, as well as their implementation prior to engaging in any aesthetic or plastic surgery procedures. This aim was to prevent the occurrence of unnecessary procedures that might compromise patients' quality of life, and to identify instances of this disease that warrant further examination.

## 2 Methods

We conducted this research in accordance with the PRISMA guidelines for systematic reviews, utilizing the MeSH terms “body dysmorphic disorder” and “prevalence.” We applied filters for original and cross‐sectional studies and conducted our search up to June 2023 in Medline via PubMed, Embase using the Ovid platform, and several Latin American databases including Scielo and TesiUNAM. We collected literature searches up to April to June 2023 and removed duplicates. Each author verified duplicates, resulting in the removal of 561 duplicated studies. Two authors screened relevant records, selecting those that met the following criteria: English and Spanish language studies with a population that included patients diagnosed with BDD and reported prevalence. Exclusion criteria included articles not related to BDD or that did not mention or describe prevalence in the article.

The following information was gathered from the chosen studies: (1) authors, (2) year of publication, (3) country, (4) sample size, (5) sample mean age (and range), (6) population size, (7) gender distribution (male and female), (8) diagnostic or screening instrument used to diagnose BDD, (9) the setting where the data were collected (plastic surgery, dermatology, psychiatric, and community), and (10) number of participants with BDD or BDD diagnosis. The data extraction was carried out by two authors to minimize bias and enhance accuracy, thus avoiding discrepancies in the collected data. An independent bias assessment was performed using the JBI critical appraisal tool to decrease the risk of bias in this systematic review. This process was carried out by each author for all included studies.

### 2.1 Statistical Analysis

The data was arranged into multiple categories for the purpose of comparing prevalence in different populations. The first group was organized by disease, which included plastic surgery, psychiatry, and dermatology, as well as community studies. Another group was based on continental locations, and the last group was designed to compare BDD diagnosis tools and BDD prevalence. This last group included MINI, YBOCS, DCQ, MBSRQ‐AS, and DQ clustered together, while BDDQ and SCID were analyzed individually. Statistical analysis was conducted using RStudio and the Meta package Version 6.5‐0, with the results of the collected database. The *p*‐value for the general sample, bias assessment, and community prevalence sample was ≤ 0.0001, while in the other subgroups it was ≤ 0.01, with heterogeneity ranging from 95% to 99%.

## 3 Results

**FIGURE 1:**
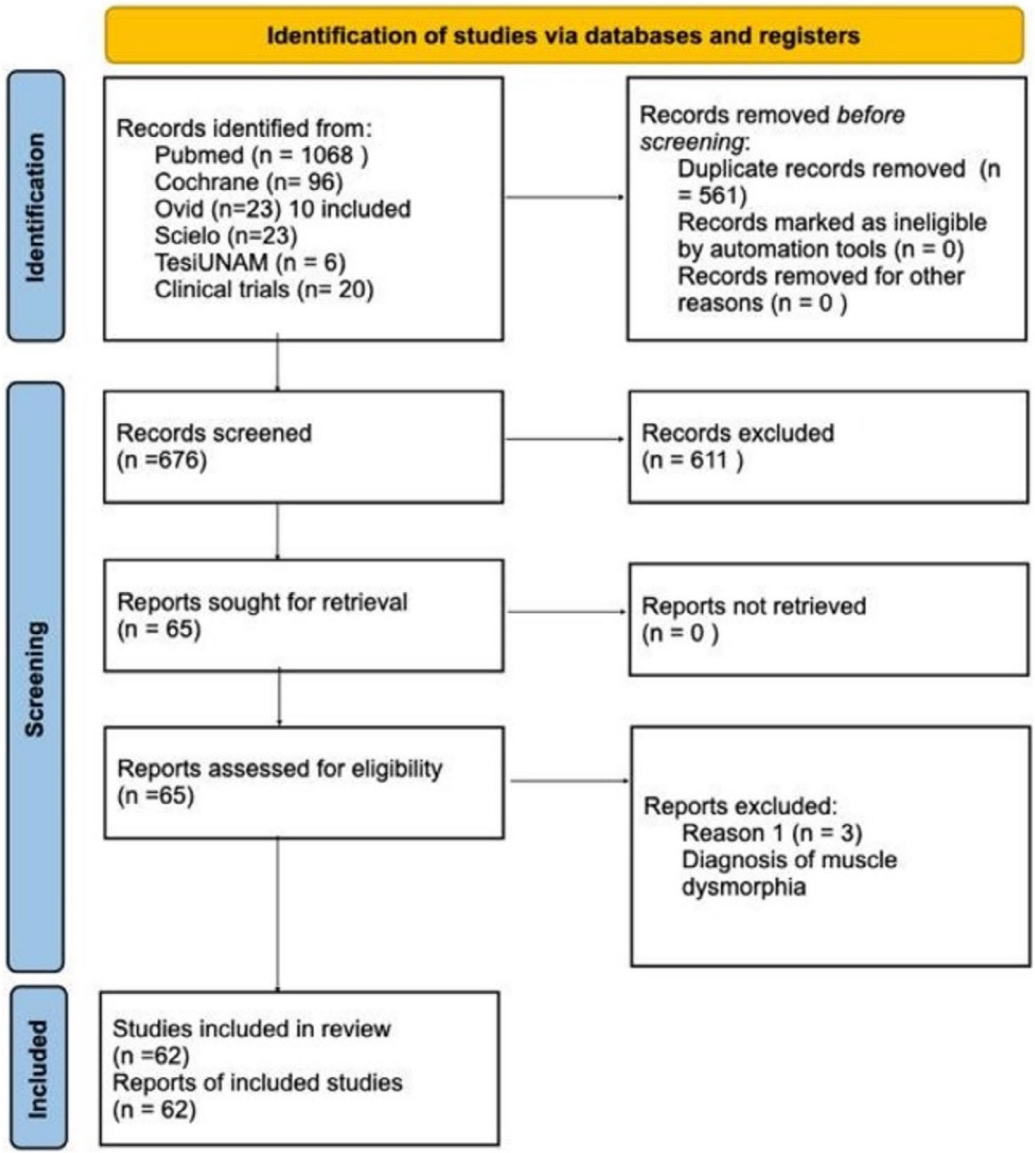
PRISMA flow diagram.

**FIGURE 2:**
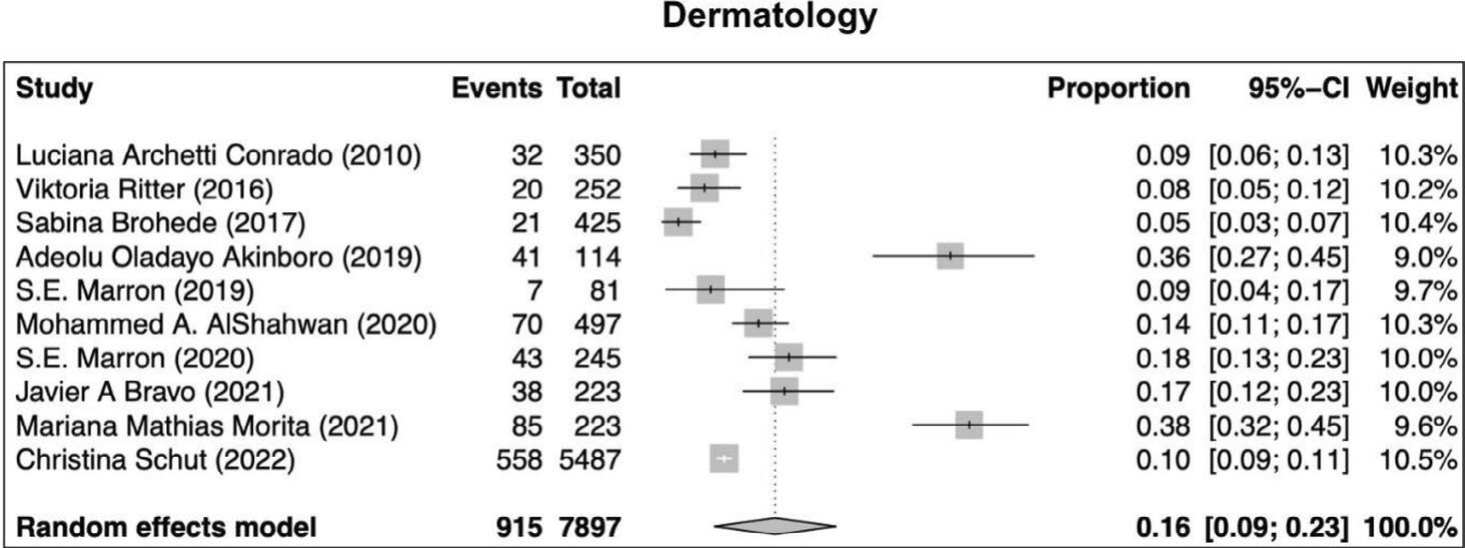
Prevalence of body dysmorphic disease in dermatology patients.

**FIGURE 3:**
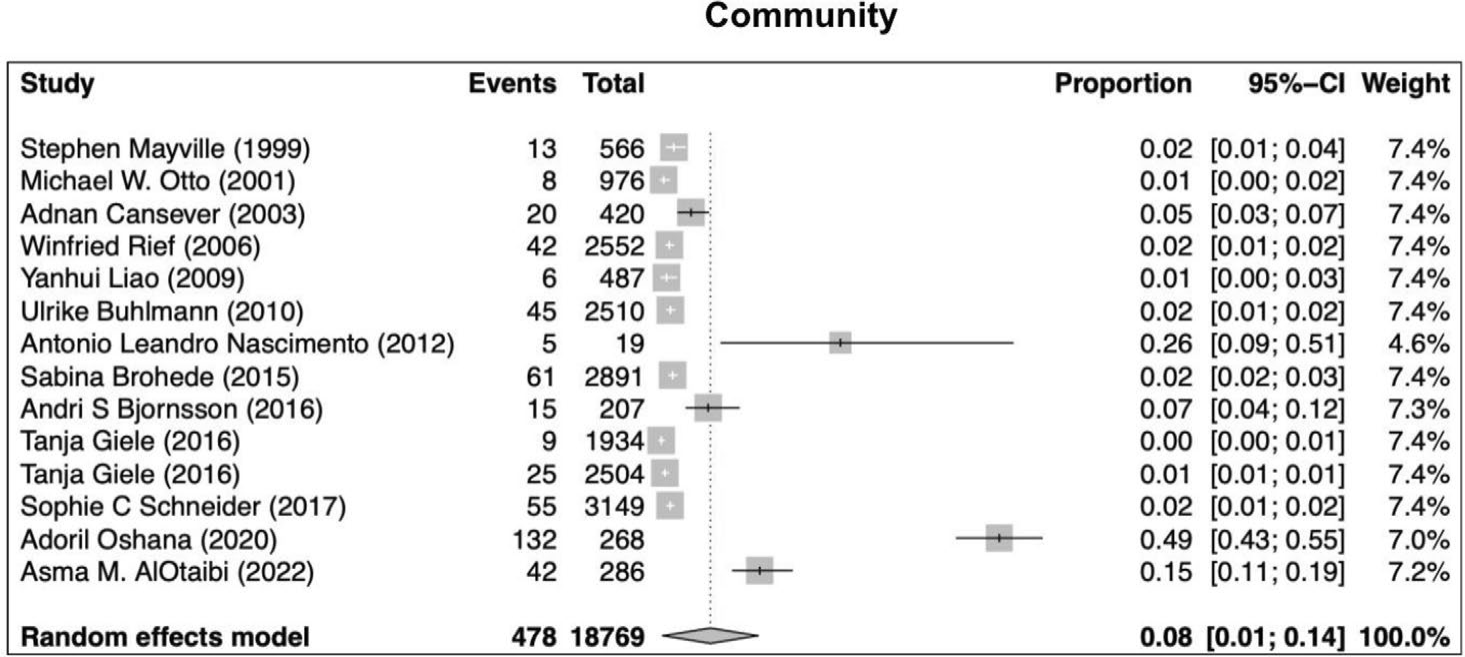
Prevalence of body dysmorphic disease in community studies.

**FIGURE 4:**
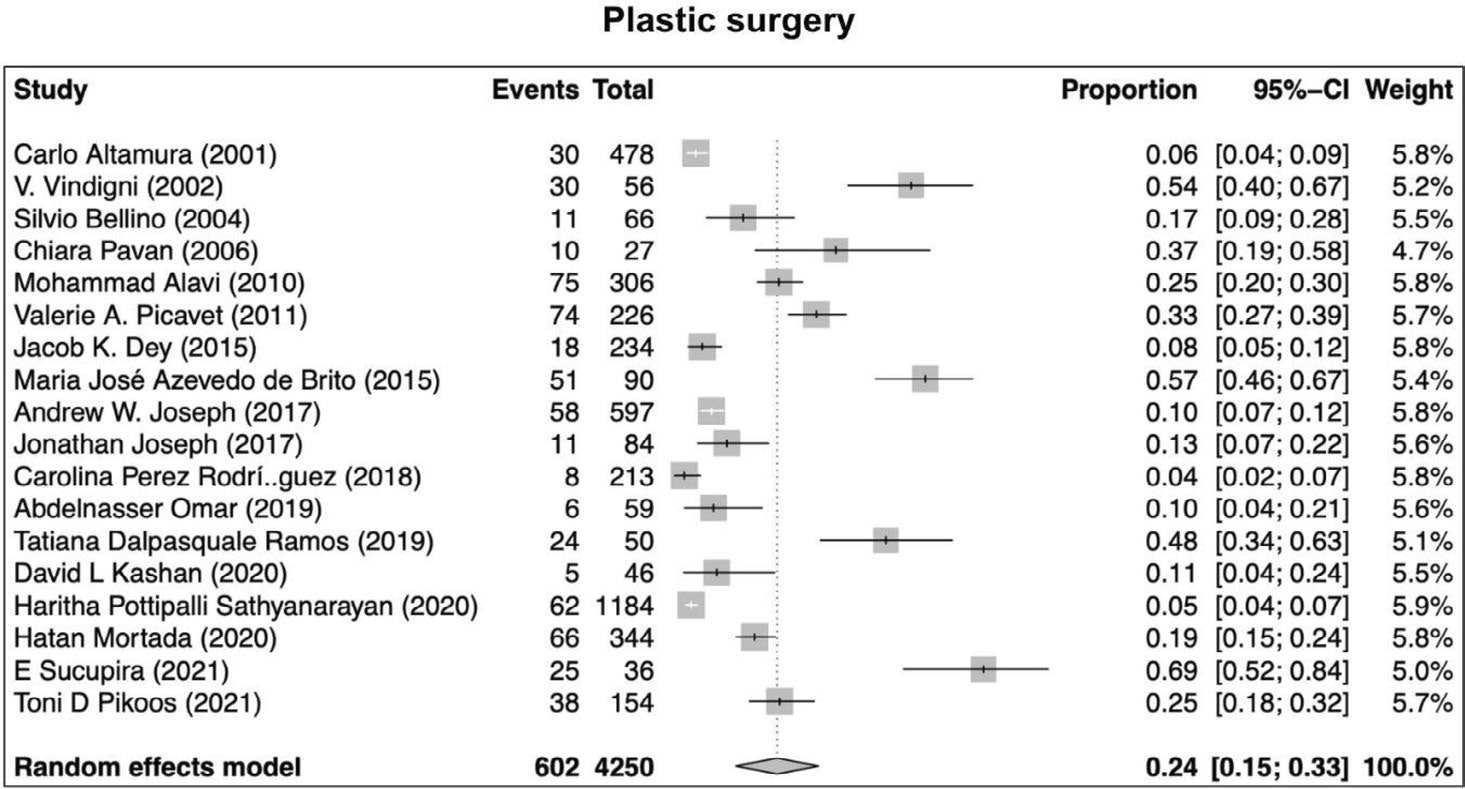
Prevalence of body dysmorphic disease in plastic surgery patients.

**FIGURE 5:**
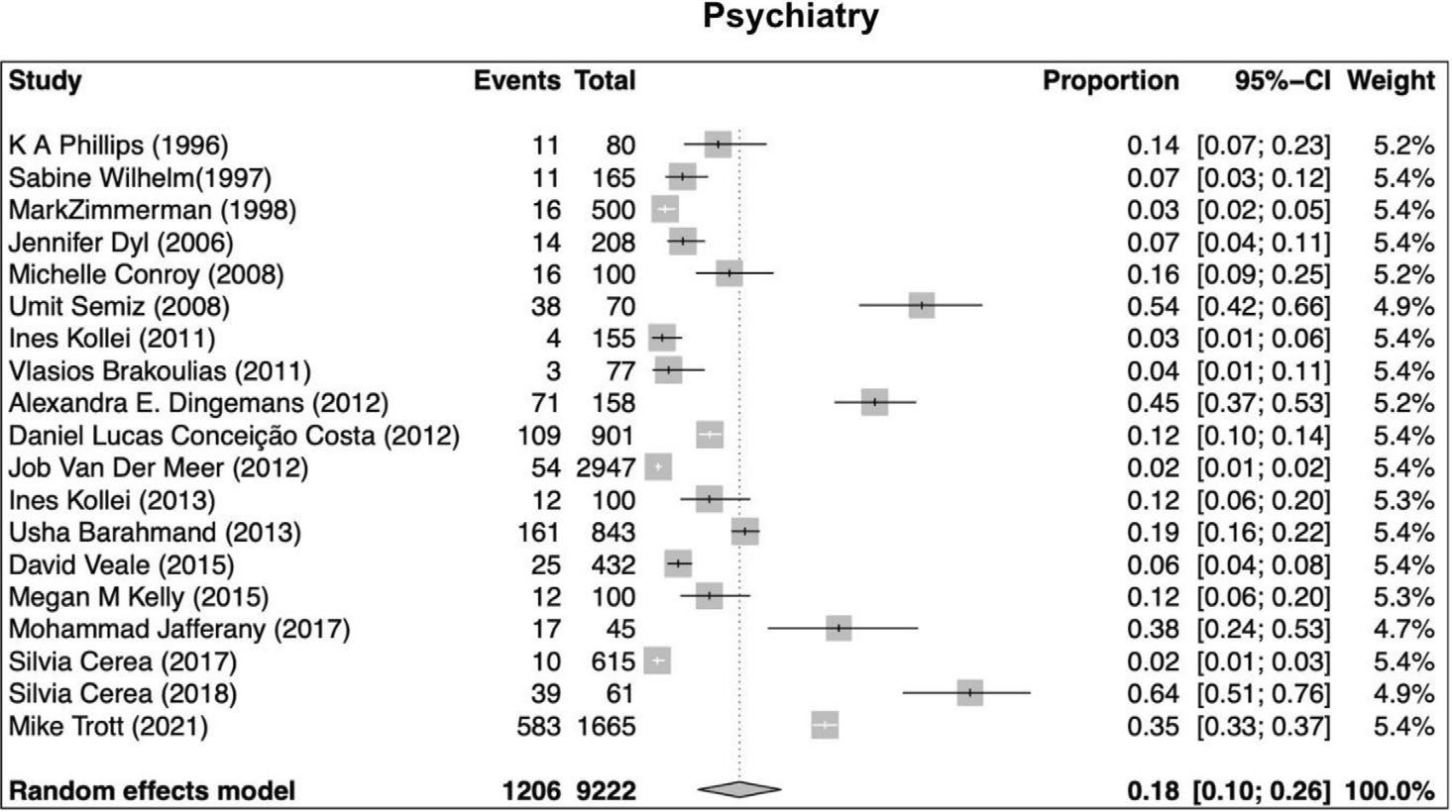
Prevalence of body dysmorphic disease in patients with psychiatric disorders.

Our search returned 1236 records, including 1068 from Pubmed, 96 from Cochrane, 10 from Ovid, 23 from Scielo, 20 from Clinical Trial, and 6 from TesiUNAM. The authors verified and removed 561 duplicate studies. Two authors screened 675 relevant records and applied selection criteria to exclude 611 records and include 65 articles. Three articles were excluded as they focused on muscle dysmorphia instead of BDD (Figure 1). We analyzed a total of 62 articles, revealing an overall incidence of BDD in the general population of 17%, with a 95% confidence interval (CI) of 13%–21%. The prevalence of BDD was found to be higher among females (16%, 95% CI 11%–21%) than males (11%, 95% CI 7%–16%). Furthermore, the prevalence was greater in patients who underwent plastic surgery (24%, 95% CI 15%–33%), followed by psychiatric patients (18%, 95% CI 10%–26%) and dermatological patients (16%, 95% CI 9%–23%) (Figures 2, 3, 4, 5).

The BDD prevalence in Latin America (31%, 95% CI 17%–46%) was found to be higher than that in North America (12%, 95% CI 5%–19%). The prevalence in other continental regions, including Asia (17%, 95% CI 7%–27%), Europe (14%, 95% CI 7%–21%), Africa (23%, 95% CI 0%–48%), and Oceania (10%, 95% CI 0%–24%), was also measured and found to be similar to that of the general population.

**FIGURE 6:**
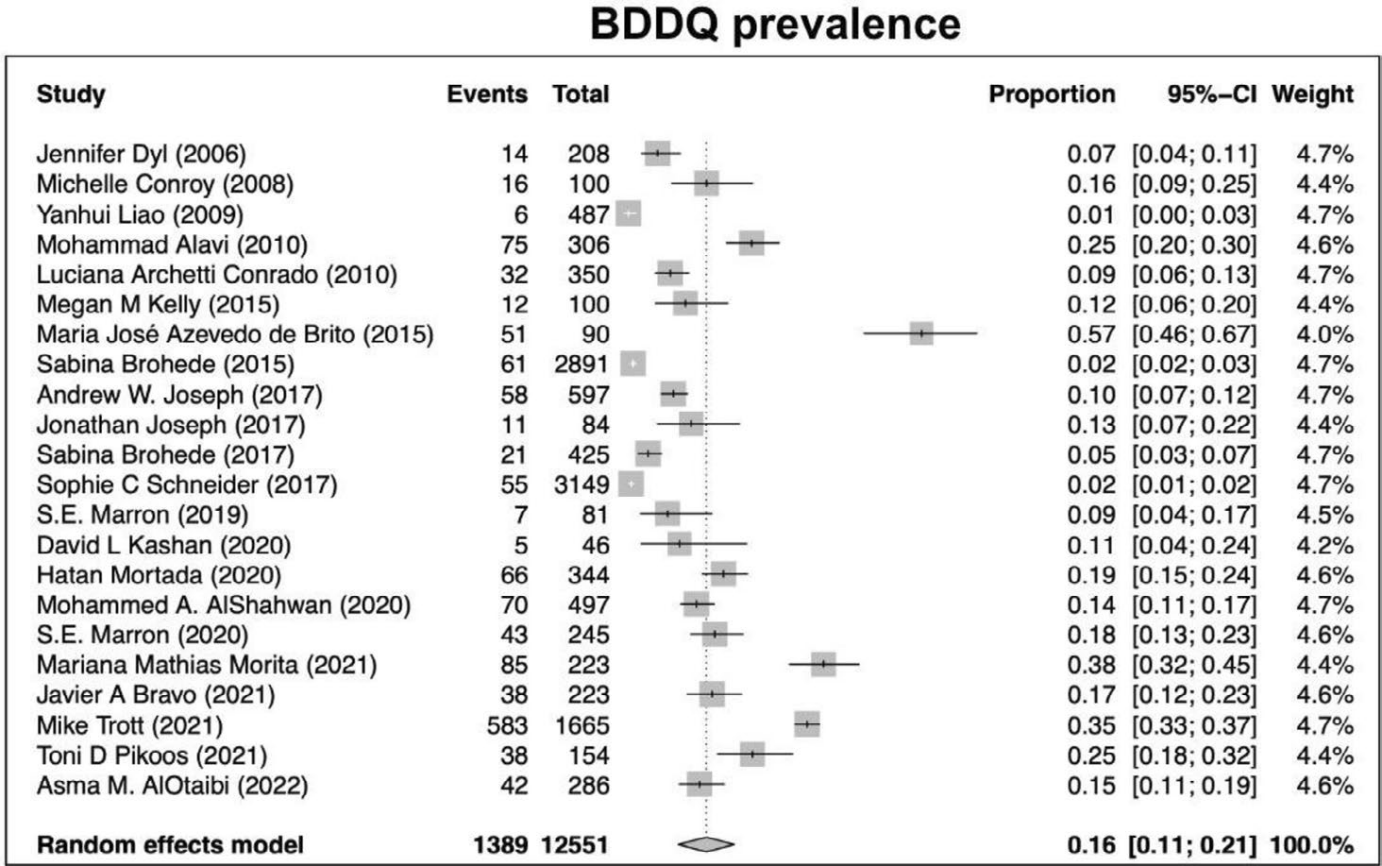
Prevalence of body dysmorphic disease in studies using the BDDQ questionnaire.

**FIGURE 7:**
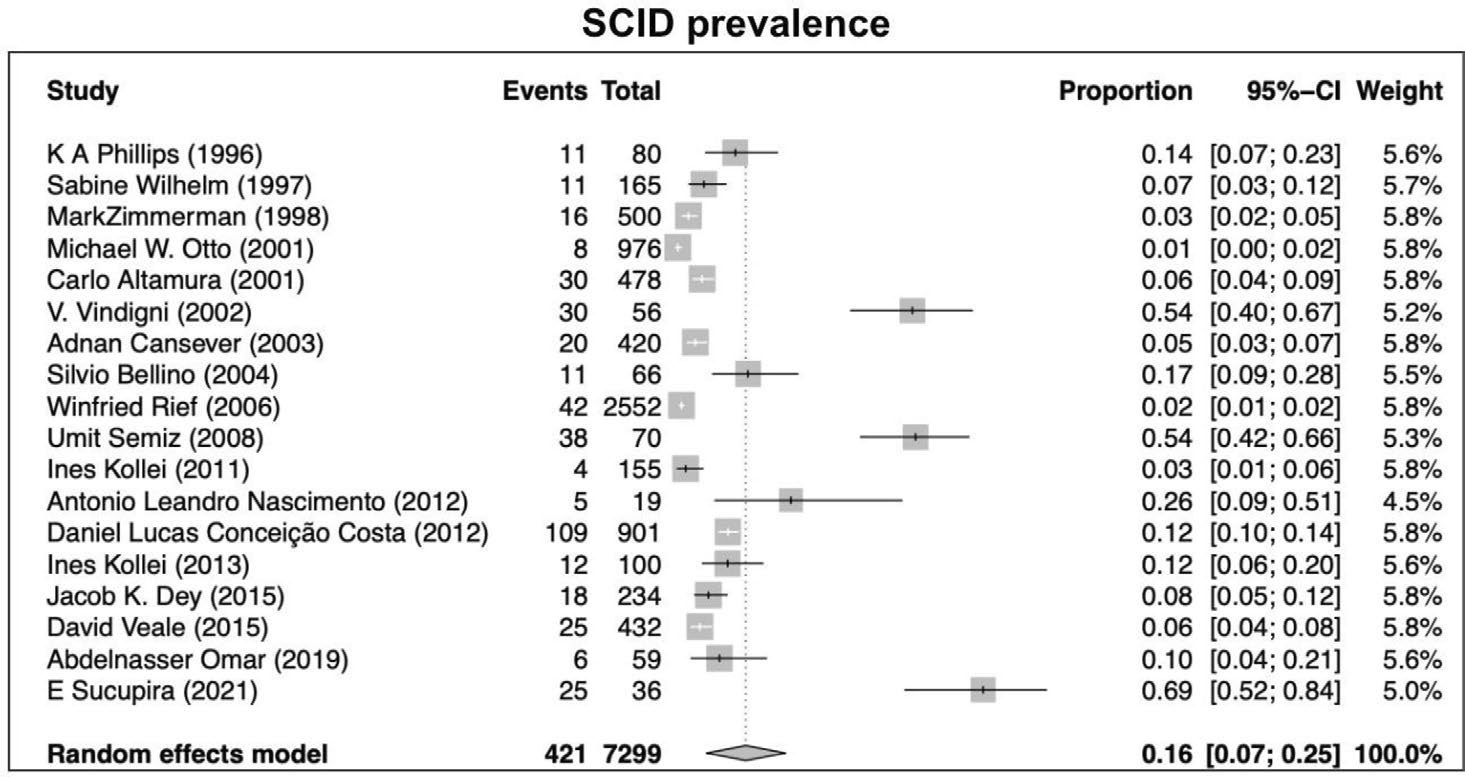
Prevalence of body dysmorphic disease in studies using the SCID.

**FIGURE 8:**
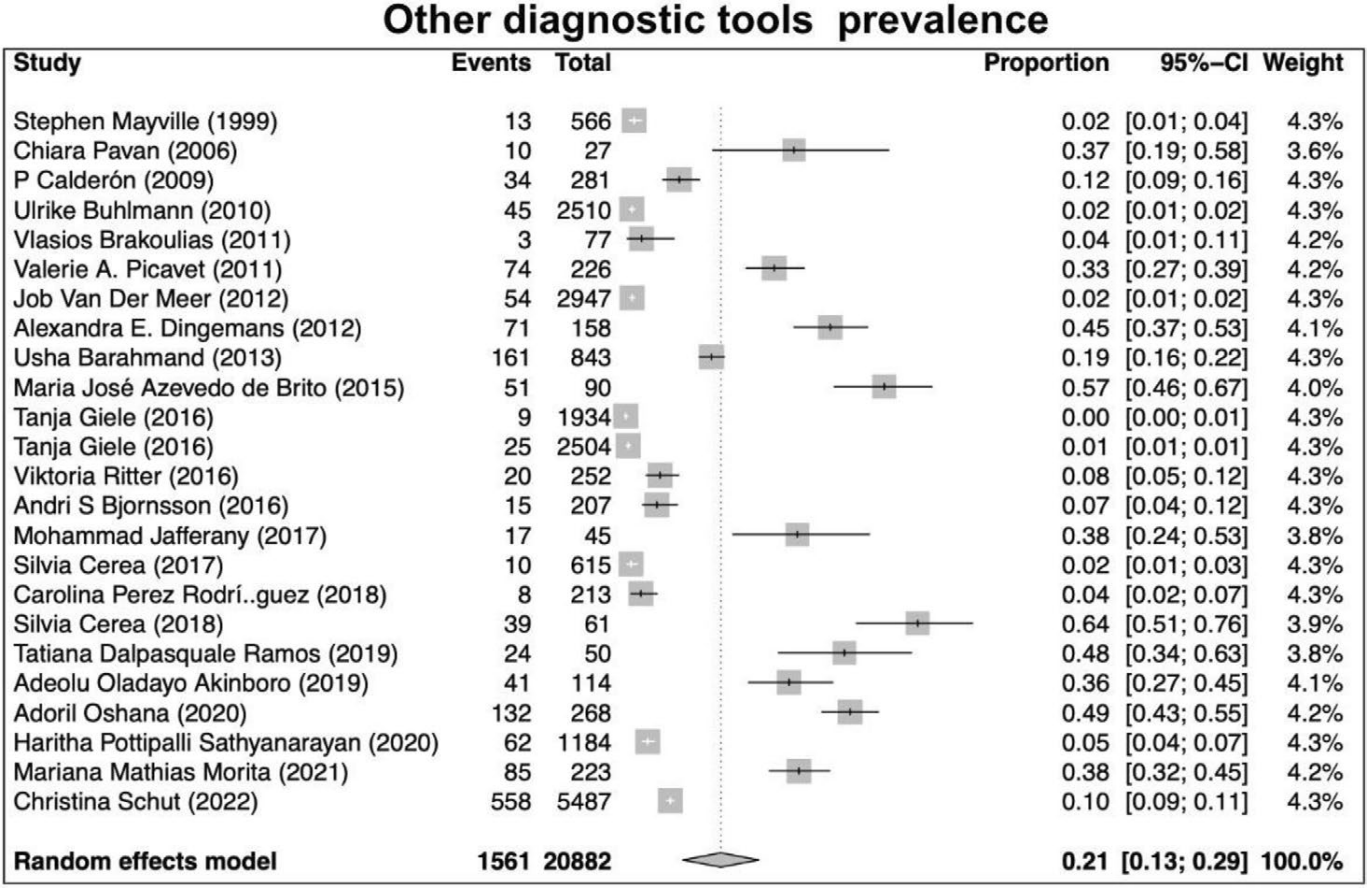
Prevalence of body dysmorphic disease in studies using other diagnostic tools: MINI, YBOCS, DCQ, MBSRQ‐AS, and DQ.

Lastly, using multiple clinical/screening tools, the highest prevalence of BDD was reported in the group using MINI, YBOCS, DCQ, MBSRQ‐AS, and DQ (21%, 95% CI 13%–29%), followed by SCID (16%, 95% CI 7%–25%), and BDDQ with the lowest prevalence (13%, 95% CI 9%–17%) (Figures 6, 7, 8).

## 4 Discussion

Body dysmorphic disorder is a mental health condition that has a considerable influence beyond the realm of psychiatry. It particularly affects individuals who undergo cosmetic procedures, as evidenced by its high prevalence among these patients. This study discovered a higher occurrence of BDD in Latin American studies compared to other regions, which aligns with the global statistics on surgical and non‐surgical cosmetic procedures. The United States reportedly leads the world in cosmetic surgeries, with over 7 million procedures performed in the country in 2022. Other Latin American countries, such as Brazil, Mexico, Argentina, and Colombia, also ranked among the top 10 countries that performed cosmetic procedures [10].

Patients who wish to undergo cosmetic surgeries often have social, psychological, and cultural factors that contribute to their decision. Research indicates that the motivation for cosmetic surgery is rooted in a mixture of psychological and emotional influences. The variation in prevalence across different geographic regions may be attributed to differences in study design, such as population‐based studies versus hospital‐based studies, as well as cultural and social factors. Although further population studies are required in countries like the United States and Latin America, and globally, to better understand this disparity, it is crucial to recognize the complex interplay of social, cultural, and psychological factors in shaping patients' decisions to undergo cosmetic procedures [11].

Among the various diagnostic instruments is the group of other diagnostic tools exhibited the highest prevalence, while the lowest prevalence was observed with the BDDQ, a self‐reported screening tool assessing preoccupations with appearance and distress using DSM‐5 criteria. Compared to the SCID, considered the gold standard diagnostic tool, the BDDQ has demonstrated high accuracy (91.7%), sensitivity (100%), and specificity (90.3%) [12, 13] Nevertheless, only 15% of studies employing the BDDQ included a psychiatric evaluation, compared to 50% for SCID and 29.2% of studies utilizing other diagnostic tools. This disparity may affect diagnostic accuracy and underscore the need for standardized psychiatric evaluation in BDD screening.

Given that individuals with BDD often experience persistent dissatisfaction regardless of cosmetic outcomes [13, 14], timely screening with tools such as BDDQ, besides a multidisciplinary approach, is crucial in cosmetic, plastic surgery, and dermatologic settings. Avoidance of unnecessary aesthetic procedures should be prioritized, integrating pharmacotherapy (primarily antidepressants) and cognitive‐behavioral therapy (CBT) to improve quality of life and mitigate suicidality risk [15, 16, 17, 18].

## 5 Conclusion

Early BDD detection ensures that patients can be timely referred to mental health professionals to provide medical and psychological treatment as needed. Cosmetic procedures should be postponed or avoided until a thorough mental health evaluation is performed, reducing unnecessary interventions and improving patient outcomes.

## Author Contributions

Morales‐Sánchez Martha Alejandra was responsible for conceptualizing the study, and both authors contributed equally to the investigation, data curation, formal analysis, validation, and writing of the manuscript. Additionally, both authors reviewed the final draft of the manuscript.

## Ethics Statement

The ethical considerations for this study did not necessitate approval from the Institutional Review Board (IRB) as it is a systematic review and meta‐analysis of existing scientific literature.

## Conflicts of Interest

The authors declare no conflicts of interest.

## Supporting information


Table S1.

## Data Availability

The authors have nothing to report.
