# Correction to “Prevalence of Body Dysmorphic Disorder: A Systematic Review and Meta‐Analysis”

**DOI:** 10.1111/jocd.70207

**Published:** 2025-04-28

Pérez‐Buenfil A , Morales‐Sánchez A . Prevalence of Body Dysmorphic Disorder: A Systematic Review and Meta‐Analysis. J Cosmet Dermatol. 2025 Apr;24(4):e70121. 10.1111/jocd.70121. PMID: 40200598; PMCID: PMC11979448.40200598 PMC11979448

There is an error in the authors' names as currently published. The correct names are:

- **Luis Angel Pérez‐Buenfil**

ORCID: https://orcid.org/0000‐0002‐9601‐4516

First Name: Luis Angel

Last Name: Pérez‐Buenfil

- **Martha Alejandra Morales‐Sánchez**

ORCID: https://orcid.org/0000‐0002‐1180‐6750

First Name: Martha Alejandra

Last Name: Morales‐Sánchez

The citation will be: **Pérez‐Buenfil LA, Morales‐Sánchez MA**. Prevalence of Body Dysmorphic Disorder: A Systematic Review and Meta‐Analysis.

We apologize for this error, and these corrections were previously submitted through the Wiley system during the proof revision stage.

