# Supplementary material for: Prevalence of Body Dysmorphic Disorder: A Systematic Review and Meta‐Analysis
Source: J Cosmet Dermatol. 2025 Apr 8;24(4):e70121. doi: 10.1111/jocd.70121 (PMC11979448; doi:10.1111/jocd.70121)
Supplement: Supplementary file 1 — Table S1. [file JOCD-24-e70121-s001.docx]

**Table 1. Studies included in the systematic review.**

| **Authors** | **Title** | **Publication year** |
| --- | --- | --- |
| Akinboro AO,Adelufosi AO,Onayemi O,Asaolu S | Body dysmorphic disorder in patients attending a dermatology clinic in Nigeria: sociodemographic and clinical correlates* | 2019 |
| Al N,Jv L,Lf. F | Body dysmorphic disorder and eating disorders in elite professional female ballet dancers | 2012 |
| Alavi M,Kalafi Y,Dehbozorgi GR,Javadpour A | Body dysmorphic disorder and other psychiatric morbidity in aesthetic rhinoplasty candidates | 2011 |
| AlOtaibi AM,Almuzaini MM,Alotaibi MA,Alotai | Prevalence and effect of body dysmorphic disorder in females at Madinah, Saudi Arabia | 2022 |
| AlShahwan MA | Prevalence and characteristics of body dysmorphic disorder in Arab dermatology patients | 2020 |
| Altamura C,Paluello MM,Mundo E,Medda S,M | Clinical and subclinical body dysmorphic disorder | 2001 |
| Barahmand U,Shahbazi Z | Prevalence of and associations between body dysmorphic concerns, obsessive beliefs and social anxiety | 2015 |
| Bellino S,Zizza M,Paradiso E,Rivarossa A,Fulche | Dysmorphic concern symptoms and personality disorders: A clinical investigation in patients seeking cosmetic surgery | 2006 |
| Bjornsson A,Magnusdottir S,Wessman I,Beard | Prevalence and Characteristics of Body Dysmorphic Disorder Among Patients in a Partial Hospital Program | 2016 |
| Brakoulias V,Starcevic V,Sammut P,Berle D,Mili | Obsessive-Compulsive Spectrum Disorders: a Comorbidity and Family History Perspective | 2011 |
| Bravo JA,Cifuentes S,Ulloa JH,Kabnick L,Pedraz | Body dysmorphic disorder in patients with telangiectasias | 2021 |
| Brohede S,Wingren G,Wijma B,Wijma K | Prevalence of body dysmorphic disorder among Swedish women: A population-based study | 2015 |
| Brohede S,Wyon Y,Wingren G,Wijma B,Wijma K | Body dysmorphic disorder in female Swedish dermatology patients | 2017 |
| Buhlmann U,Glaesmer H,Mewes R,Fama JM,W | Updates on the prevalence of body dysmorphic disorder: A population-based survey | 2010 |
| Calderón P,Zemelman V,Sanhueza P,Castrillón | Prevalence of body dysmorphic disorder in Chilean dermatological patients | 2009 |
| Cansever A,Uzun Ö,Dönmez E,Özşahin A | The prevalence and clinical features of body dysmorphic disorder in college students: A study in a Turkish sample | 2003 |
| Cerea S,Bottesi G,Grisham JR,Ghisi M | Body dysmorphic disorder and its associated psychological and psychopathological features in an Italian community sample | 2018 |
| Cerea S,Bottesi G,Grisham JR,Ghisi M | Non-weight-related body image concerns and Body Dysmorphic Disorder prevalence in patients with Anorexia Nervosa | 2018 |
| Conrado LA,Hounie AG,Diniz JB,Fossaluza V,Tor | Body dysmorphic disorder among dermatologic patients: Prevalence and clinical features | 2010 |
| Conroy M,Menard W,Fleming-Ives K,Modha P,C | Prevalence and clinical characteristics of body dysmorphic disorder in an adult inpatient setting | 2008 |
| Costa DL,Assunção MC,Ferrão YA,Conrado LA,G | BODY DYSMORPHIC DISORDER IN PATIENTS WITH OBSESSIVE–COMPULSIVE DISORDER: PREVALENCE AND CLINICAL CORRELATES | 2012 |
| de Brito MJ,Nahas FX,Cordás TA,Gama MG,Suc | Prevalence of Body Dysmorphic Disorder Symptoms and Body Weight Concerns in Patients Seeking Abdominoplasty | 2016 |
| Dey JK,Ishii M,Phillis M,Byrne PJ,Boahene KD,Is | Body dysmorphic disorder in a facial plastic and reconstructive surgery clinic: measuring prevalence, assessing comorbidities, and validating a feasible screening instrument | 2015 |
| Dingemans AE,van Rood YR,de Groot I,van Furt | Body dysmorphic disorder in patients with an eating disorder: Prevalence and characteristics | 2012 |
| Dyl J,Kittler J,Phillips KA,Hunt JI | Body Dysmorphic Disorder and Other Clinically Significant Body Image Concerns in Adolescent Psychiatric Inpatients: Prevalence and Clinical Characteristics | 2006 |
| Gieler T,Schmutzer G,Braehler E,Schut C,Peters | Shadows of Beauty – Prevalence of Body Dysmorphic Concerns in Germany is Increasing: Data from Two Representative Samples from 2002 and 2013 | 2016 |
| Jafferany M,Osuagwu FC,Khalid Z,Oberbarnsch | Prevalence and clinical characteristics of body dysmorphic disorder in adolescent inpatient psychiatric patients—a pilot study | 2019 |
| Joseph AW,Ishii L,Joseph SS,Smith JI,Su P,Bater | Prevalence of Body Dysmorphic Disorder and Surgeon Diagnostic Accuracy in Facial Plastic and Oculoplastic Surgery Clinics | 2017 |
| Joseph J,Randhawa P,Hannan SA,Long J,Goh S, | Body dysmorphic disorder in patients undergoing septorhinoplasty surgery: should we be performing routine screening? | 2017 |
| Kashan DL,Horan MP,Wenzinger E,Kashan RS,B | Identification of Body Dysmorphic Disorder in Patients Seeking Corrective Procedures From Oral and Maxillofacial Surgeons | 2021 |
| Kelly MM,Zhang J,Phillips KA | The prevalence of body dysmorphic disorder and its clinical correlates in a VA primary care behavioral health clinic | 2015 |
| Kollei I,Martin A,Rein K,Rotter A,Jacobi A,Muel | Prevalence of body dysmorphic disorder in a German psychiatric inpatient sample | 2011 |
| Kollei I,Schieber K,de Zwaan M,Svitak M,Martin | Body dysmorphic disorder and nonweight-related body image concerns in individuals with eating disorders | 2013 |
| Liao Y,Knoesen NP,Deng Y,Tang J,Castle DJ,Book | Body dysmorphic disorder, social anxiety and depressive symptoms in Chinese medical students | 2010 |
| Marron SE,Gracia-Cazaña T,Miranda-Sivelo A,L | Screening for Body Dysmorphic Disorders in Acne Patients: A Pilot Study | 2019 |
| Marron SE,Miranda-Sivelo A,Tomas-Aragones L | Body dysmorphic disorder in patients with acne: a multicentre study | 2020 |
| Mayville S,Katz RC,Gipson MT,Cabral K | Assessing the Prevalence of Body Dysmorphic Disorder in an Ethnically Diverse Group of Adolescents | 1999 |
| Morita MM,Merlotto MR,Dantas CL,Olivetti FH | Prevalence and factors associated with body dysmorphic disorder in women under dermatological care at a Brazilian public institution | 2021 |
| Mortada H,Seraj H,Bokhari A | Screening for body dysmorphic disorder among patients pursuing cosmetic surgeries in Saudi Arabia | 2020 |
| Omar A,Eid M,Ali R,Missiry ME,Gawad AA,Gho | Psychiatric morbidity among Egyptian patients seeking rhinoplasty | 2019 |
| Oshana A,Klimek P,Blashill AJ | Minority stress and body dysmorphic disorder symptoms among sexual minority adolescents and adult men | 2020 |
| Otto MW,Wilhelm S,Cohen LS,Harlow BL | Prevalence of Body Dysmorphic Disorder in a Community Sample of Women | 2001 |
| Pavan C,Vindigni V,Semenzin M,Mazzoleni F,Ga | Personality, temperament and clinical scales in an Italian Plastic Surgery setting: what about body dysmorphic disorder? | 2006 |
| Phillips KA,A. NA,Brendel,G. F | Prevalence and Clinical Features of Body Dysmorphic Disorder in Atypical Major Depression | 1996 |
| Picavet VA,Prokopakis EP,Gabriëls L,Jorissen M | High Prevalence of Body Dysmorphic Disorder Symptoms in Patients Seeking Rhinoplasty | 2011 |
| Pikoos TD,Rossell SL,Tzimas N,Buzwell S | Is the needle as risky as the knife? The prevalence and risks of body dysmorphic disorder in women undertaking minor cosmetic procedures | 2021 |
| Ramos TD,de Brito MJ,Suzuki VY,Neto MS,Ferre | High Prevalence of Body Dysmorphic Disorder and Moderate to Severe Appearance-Related Obsessive–Compulsive Symptoms Among Rhinoplasty Candidates | 2019 |
| Rief W,Buhlmann U,Wilhelm S,Borkenhagen AD | The prevalence of body dysmorphic disorder: a population-based survey | 2006 |
| Ritter V,Fluhr JW,Schliemann-Willers S,Elsner P, | Body dysmorphic concerns, social adaptation, and motivation for psychotherapeutic support in dermatological outpatients | 2016 |
| Rodríguez CP,Judge RB,Castle D,Phillipou A | Body dysmorphia in dentistry and prosthodontics: A practice based study | 2019 |
| Sathyanarayana HP,Padmanabhan S,Balakrishn | Prevalence of Body Dysmorphic Disorder among patients seeking orthodontic treatment | 2020 |
| Schneider SC,Turner CM,Mond J,Hudson JL | Prevalence and correlates of body dysmorphic disorder in a community sample of adolescents | 2016 |
| Schut C,Dalgard FJ,Bewley A,Evers AW,Gieler U | Body dysmorphia in common skin diseases: results of an observational, cross-sectional multicentre study among dermatological outpatients in 17 European countries* | 2022 |
| Semiz U,Basoglu C,Cetin M,Ebrinc S,Uzun O,Erg | Body dysmorphic disorder in patients with borderline personality disorder: prevalence, clinical characteristics, and role of childhood trauma | 2008 |
| Sucupira E,Brito MJ,Leite AT,Aihara EM,Neto M | Body dysmorphic disorder and personality in breast augmentation: The big-five personality traits and BDD symptoms | 2022 |
| Trott M,Johnstone J,Firth J,Grabovac I,McDerm | Prevalence and correlates of body dysmorphic disorder in health club users in the presence vs absence of eating disorder symptomology | 2021 |
| van der Meer J,van Rood YR,van der Wee NJ,de | Prevalence, demographic and clinical characteristics of body dysmorphic disorder among psychiatric outpatients with mood, anxiety or somatoform disorders | 2012 |
| Veale D,Akyüz EU,Hodsoll J | Prevalence of body dysmorphic disorder on a psychiatric inpatient ward and the value of a screening question | 2015 |
| Vindigni V,Pavan C,Semenzin M,Granà S,Gamb | The importance of recognizing body dysmorphic disorder in cosmetic surgery patients: do our patients need a preoperative psychiatric evaluation? | 2002 |
| Wilhelm S,Otto MW,Zucker BG,Pollack MH | Prevalence of Body Dysmorphic Disorder in patients with anxiety disorders | 1997 |
| Zimmerman M,Mattia JI | Body dysmorphic disorder in psychiatric outpatients: Recognition, prevalence, comorbidity, demographic, and clinical correlates | 1998 |
